# What are the greatest opportunities for innovation to improve access to and quality of palliative care services to children? A qualitative interview study

**DOI:** 10.1186/s12904-025-01837-9

**Published:** 2025-07-02

**Authors:** K. Grailey, J. Roland, M. Lambert, G. Fontana, L. Dale-Harris, I. Williams, A. Shaw, A. L. Neves, J. Downing

**Affiliations:** 1 Centre for Health Policy, Institute of Global Health Innovation, Imperial College London, Room1034 10th Floor QEQM Building, St Mary’s Campus, Paddington, London, W2 1NY UK; 2 Department of Primary Care and Public Health, Imperial College London, London, UK; 3 Helix Centre, Institute of Global Health Innovation, Imperial College London, London, UK; 4 Global Treehouse Foundation, London, UK; 5 International Children’s Palliative Care Network, Bristol, UK

**Keywords:** Innovation, Children’s palliative care, Qualitative research, Paediatric palliative care, Global health, Human centred design, Child health, Person centred care

## Abstract

**Background:**

Innovation is required within children’s palliative care (CPC) to improve access to and quality of care. Existing research demonstrates a lack of access to CPC worldwide. This study aimed to explore the current opportunities for innovation within CPC from the provider’s perspective.

**Methods:**

Global leaders in CPC were purposively sampled and invited to partake in a qualitative semi-structured interview. Data were thematically analysed using an inductive approach.

**Results:**

Fifty-one CPC leaders from 27 countries were interviewed, encompassing clinical, managerial and patient advocacy roles. A thematic framework housing nine key themes was created, reflecting what innovation means in the context of CPC and the future of innovation. Key findings included opportunities to leverage new technologies within the sector. The importance of a clear vision and user-centred design were seen as crucial when innovating in CPC.

**Conclusions:**

This study provides novel insights into opportunities for innovation in CPC, creating a thematic framework that brings together key themes and may help guide future innovation. We also highlight essential areas for innovation such as the use of technology. In the context of existing guidance for the delivery of CPC, leaders and policy makers can use the insights from this work to innovate and improve quality and access to CPC worldwide.

**Supplementary Information:**

The online version contains supplementary material available at 10.1186/s12904-025-01837-9.

## Introduction

The provision of children’s palliative care (CPC), required for “life-threatening conditions for which curative treatment may be feasible but can fail”, “conditions where premature death is inevitable”, “progressive conditions without curative treatment options” or “irreversible but non-progressive conditions causing severe disability leading to susceptibility to health complications and likelihood of premature death”, is essential to ensure the best possible holistic care for affected children and their families [1]. The need for this care is significant – in 2015 more than 21 million children were estimated as needing access to palliative care services across the globe [1, 2].

However, despite this need, research has demonstrated a clear lack of access to CPC in many countries. A 2011 systematic review identified that 65.5% of countries had no known children’s palliative care provision at that time [3]. Work in Kenya, South Africa and Zimbabwe demonstrated that less than 1–5% of children needing palliative care could access it [4, 5]. The need is also disproportionate across the globe - there is evidence to suggest that up to 98% of children who need palliative care live in low- and middle-income countries (LMICs) [6].

The disparity between the need for and provision of CPC is not limited to LMICs - in 2012/13 only around 10–20% of children in the US who needed palliative care were receiving it [7, 8]. Likewise in Europe it has been estimated that each year 170,000 children who would benefit from CPC die without access to it [9]. This research has led to the global estimation of less than 5% of children who need CPC are able to access it [2, 10].

There are many features of CPC that make it a unique service, when compared to other healthcare provisions. These include the need for bespoke communication methods appropriate for children, different principles surrounding consent and assent, changes to medication doses, accommodating the needs of children of different ages, and the need to adapt care as children transition into adulthood [11]. Additionally, the range of conditions for which children need palliative care is broad, children often receive palliative care for many years [12–14], and some have rare diseases of which they may be only one of a few sufferers worldwide [15].

There is an urgent need for improvement in the provision of CPC. To reach all children in need with quality palliative care services, significant innovation within the sector is required. This innovation is needed both in the context of limited funding to improve patient experience and efficiency of the service, but also to improve the nature and provision of funding itself.

Proposed areas for improvement include better training for health workers, increasing the availability of palliative care medications such as opiates for pain management, integration into existing health systems, increasing the evidence base and the creation of more accurate data collection mechanisms. It is also important to improve the physical access to CPC, thereby limiting the need for the displacement of families in order to access care [16].

The current lack of funding and recognition of the need for CPC contributes to a disparity in the provision of care [17, 18].Globally, there is limited government funding allocated to improving and innovating CPC services. As such, the third sector (including charities and philanthropic organisations) is often found attempting to fill the resulting gap [13].

Effective CPC requires a holistic approach involving multiple healthcare services and widespread community support from a range of sources. The provision of good quality CPC is multifaceted, and must address the social, emotional, physical and spiritual needs of the child and their family. The service must be adaptable and have the flexibility to be implemented in a number of settings – including the home, the community, health centres, hospitals and tertiary facilities [5]. Furthermore, it is critical to involve families in decision making and provision of care – knowing what to expect has been reported as a key priority for families [19]. It is important that care is provided where the child and family prefer it to be - this may be at home, with respite care or in hospitals [20].

There is evidence of the emergence of new, innovative models of care and education within CPC which could be replicated across the world [21]. This could increase access and enable the delivery of more consistent and person-centred, holistic, CPC globally [22–24]. Innovation in CPC is an important area to investigate because demand for CPC services is rapidly rising [14]. Action needs to be taken to make care more accessible globally in different settings, each with variable resources.

This study aimed to explore the current opportunities for innovation within CPC from the perspective of the provider, with two specific research objectives; (a) What does innovation mean in the context of CPC? and (b) What does the future of innovation in CPC look like?

## Methods

### Study design

This study was designed as a qualitative study using semi-structured interviews to explore the research aim and objectives. This manuscript is reported in line with the Standards for Reporting Qualitative Research (SRQR) guidelines [25]. The associated checklist can be viewed in Supplementary File 1.

### Terminology

For the purposes of this study the following definitions were used:

A ***child*** is defined as any person under the age of 18 (United Nations [26]) i.e. babies, toddlers, children and adolescents.

***Children’s palliative care (CPC)*** is defined in line with the WHO definition such that “palliative care for children is the active total care of the child’s body, mind and spirit and also involves giving support to the family. It begins when illness is diagnosed, and continues regardless of whether or not a child received treatment directed at the disease.” [27] It is provided for babies, children and young people under the age of 18 years.

***Innovation*** is defined broadly in line with the WHO definition [28] as encompassing the novel creation, adoption or adaption of policies, practices, systems, technologies, services and delivery methods within CPC, to improve the efficiency, effectiveness, quality, safety or affordability of the care available.

### Study setting

To address the study aim, participants were chosen who were CPC leaders with broad global distribution in order to collate a wide range of perspectives on the opportunities for innovation worldwide.

### Participants

Participants were purposively sampled using recommendations by global CPC leaders and recruited based on the following inclusion criteria:

- Viewed by their professional peers as CPC leaders from around the world e.g. service providers, senior managers, advocates, or CPC leaders were defined as those in professional positions of seniority.
- Those who had expert knowledge of innovation in the healthcare setting and how these are translated and adopted at scale.
- Were reachable by email and able to undertake a virtual interview.
- That they represented a broad global distribution representative of those working in CPC.
- Were able to conduct the interview in English.

Participants were given an information sheet prior to commencement of the study and provided written informed consent. Participants were free to withdraw from the study at any time. Recruitment ceased when thematic saturation of the qualitative data was deemed to have been achieved by the interviewing team (JR, ML, AS). No children under the age of 18 were directly involved in the study.

### Data collection and processing

A semi-structured interview guide was created for this study to explore the research objectives (Supplementary File 2). The interview guide for the semi-structured interviews was developed through a targeted literature review focusing around three key areas: (a) The current state of and global unmet need for CPC; (b) current best practice and trends among CPC providers to address these needs; and (c) reports on the wide future of health care service delivery and health innovation globally that might have relevance to the CPC sector.

As such, the interview guide covered the following domains: the progression of CPC over the last several decades, changes in the provision of services from hospital based to home based, the provision of respite care for families, the evolution of mental health support, experiences of care integration of palliative care services and experiences of providing care, and the most promising areas of future innovation and improvement in CPC. It was designed to capture current observations on these topics, alongside participants’ aspiration for how these might evolve, innovate and scale in the future. As the interviews progressed, the guide was iterated to reflect the content and emerging themes of preceding interviews. Participant demographic data were also collected (professional role, age, ethnicity, geographic location and gender). Interviews took place virtually over the Zoom platform (Zoom video communications incorporated) and were planned for up to 60 min. Interviews were audio-recorded via this platform and transcribed prior to analysis.

### Data analysis

Anonymised interview transcripts were analysed according to a thematic analysis approach to identify common themes and address the two research objectives. In accordance with the methodology proposed by Braun & Clarke [29] this took place following the sequential steps of familiarisation with the data sets, generation of initial codes, re-review of the entire dataset and grouping these codes into themes. Themes were refined and organised, ultimately leading to the construction of a thematic framework. This was a recursive process, with the codes/themes developed at each stage being reviewed against previous iterations and refined through discussion with the wider research team.

Interview transcripts were analysed by one researcher (ML), with over 90% reviewed and analysed by a second researcher (JR) for concordance and agreement in the developing themes. Any discrepancies between researchers were resolved through discussion with the wider study team.

### Researcher characteristics and reflexivity

The research team was multidisciplinary and included expertise in research, design, innovation and healthcare. This diversity within the team allowed for collaboration and communication throughout the study and provided an awareness of potential researcher bias. The study team were aware of their assumptions towards the need for ongoing innovation in children’s palliative care prior to the start of the interview process. To mitigate this, regular reflexive discussion took place throughout the interview and analysis process, supported using semi-structured topic guides to steer each interview conversation.

## Results

### Participants characteristics

Fifty-one individuals across 27 countries and six continents participated in the study. Five participants worked in clinical roles related to CPC, 13 held CPC related managerial roles and 24 worked in a mixed clinical / managerial CPC post. Seven participants were well known advocates for CPC globally, and three participants were innovation experts. At least two of the advocates included in the participant group were also family members of a child who had received palliative care.

### Qualitative interviews

Interviews ranged between 20 and 95 min, with an average duration of 47 min. The inductive thematic analysis resulted in the creation of a thematic framework housing nine broad themes that reflected the current opportunities for innovation within CPC, with most themes encompassing both research objectives of what innovation means in the context of CPC, and what the future of innovation in CPC looks like. These nine themes housed 22 subthemes (Table 1).

**Table 1:** Thematic framework housing identified opportunities for innovation in children’s palliative care

Theme	Sub-Theme
Vision	Provision of participatory mechanisms
	Capture views of all population in need
	Ambitions to grow
Services	Integrated service arrangements
	Psycho-spiritual-social support services
People	Porous teams
	Supporting staff wellbeing
	Families as part of the workforce
Technology	Access to specialist expertise
	Data driven care
	Remote delivery
Organisation	Organisational Models
Culture	Support for frontline innovation
	Key ingredients of systematic innovation culture
Place	De-medicalising the healthcare environment
	Applying design-thinking
	Changing role of the physical space
Partnerships	Community assets
	Strategic partnerships
Leadership	Advocacy
	Education
	Research

#### Vision

Having a clear vision for the provision of CPC, co-designed with service providers, children and their families was a strong theme within the data. Within this were three subthemes, each reflecting an aspect of creating a vision for CPC provision that kept shared decision making, personalised care and patient empowerment at its centre. Participants reflected upon the need for the provision of participatory mechanisms, such as consultations with children/families, or their inclusion in governance.

*And right from the start*,* we also had parents on our regional steering committee…we had a parent’s advisory committee. So I think it’s critically important to always have parents involvement in formal ways and informal ways. (Participant 15*,* Manager)*

It was important to participants that the views and needs of all those in need of CPC were captured and reflected in how such care was provided.

*That is the most important thing. Listen to the children*,* listen to the families*,* they will voice their needs. And that informed every step that that we took right up to the design of [our organisation]*,* every part of [our organisation] is based on a stated wish or need of the children and their families. (Participant 3*,* Manager)*

Finally, there was a strong sense that CPC providers should have strong ambitions to grow their service, responding directly to an increasingly growing and diversifying need.

*we sat down and created a new longer-term plan for the future that was ambitious and was based on various assumptions around being able to invest more money. But the starting point of it was talking to families and really talking and listening. (Participant 41*,* Manager)*

#### Services

Participants discussed the complexity of delivering CPC. The requirement for seamless care that integrates the full spectrum of services (including clinical care, psychological/wellbeing support and access to the multidisciplinary team) being provided was a strong theme within the data – highlighting the need for collaboration to deliver the best possible service for users. Participants also described the need for the provision of holistic care (incorporating physical, psychological, social and spiritual support to both children and their families), as well as support from the CPC provider as families navigate these multiple offerings.

*We call it the managed clinical network. [hospice] centres*,* symptom management and nursing services…counselling art therapy*,* music therapy*,* community events… A rota of 6 [clinicians] employed by their own agencies but who agree it is part of their work to care for these children through the hospice. Completely across the clinical path. Only accessed through the clinical nurse specialist.(Participant 46*,* Clinician/ Manager)*

*You talk about innovation*,* it’s really very simple. People have to work in partnership to achieve this and you don’t*,* you can’t have a team of staff sitting around waiting for children to die and need that support. You’ve got to have an infrastructure in place that enables that integrated model of care and that collaborative working across systems because you will need you know the territory consultant who is an expert in symptom management*,* you will need the local children’s community nurse who knows the family and has been there from the outset. (Participant 52*,* Clinician/ Manager)*

#### People

The importance of the team delivering CPC, and providing adequate support to the team when delivering potentially challenging work was an important theme within the data. As highlighted in the previous theme, the need for an increasing diversity of offering within CPC, the resultant need for specialisation and increased skill mix, and the requirement for integrated care for the user led to participants describing a need for ‘porous’ teams – where external services work harmoniously with core CPC providers. It was also highlighted that families of children often become part of the workforce, especially given that CPC is often delivered at home.

*[Our] priority is to truly integrate and collaborate as teams (hospital*,* hospice*,* community and primary care*,* third sector)*,* advancing together towards a shared goal and plan*,* without leaving parts of the system behind. Sharing of information is key (Participant 11*,* Clinician/ Manager)*

*complexity of care that children require is much greater and more and more of that’s being done at home. There isn’t a workforce that’s equipped to deliver a lot of that care out. And that’s why families are doing more of it at home (Participant 24*,* Manager)*

The requirement for support for staff and their wellbeing was a prominent sub-theme, with participants reflecting upon the unique stresses of delivering CPC, and the need for support to reduce burnout and remain compassionate.

*Staff wellbeing is absolutely vital. And I would say this for all employers in all sectors*,* but it’s certainly true in palliative care where you are going to see children dying… We set up a confidential mental health counselling helpline for all staff*,* and for all members of their immediate family as well*,* if they were concerned about the member of staff. (Participant 21*,* Clinician/ Manager)*

#### Technology

The utilisation of digital technology and how it can be harnessed to innovate and develop the future provision of CPC was prominent within the dataset. Participants described using digital technology to facilitate the remote delivery of care, allowing better access to a limited resource (especially over large geographical areas), and how this minimised barriers faced due to distance or mobility challenges.

*That is so helpful*,* not only for families to keep their child at home*,* but for the local workers to help the family keep their child at home… You can do it without being in person. Yeah*,* no doubt*,* the whole idea of telemedicine*,* I think*,* is a really strong aspect of…extending palliative care and hospice services over distances. (Participant 15*,* Manager)*

Data-driven care was highlighted by participants as crucial, requiring investment in systems and ensuring the team had the right skills to deliver this.

*[We realised we needed someone with a portfolio in terms of data and key Performance Indicators and strategy. So*,* the world is our oyster*,* as they say… our data field is expanding on a daily basis in terms of statistics*,* in terms of mining and all that so it will be fascinating to see what’s what comes next in the next few years (Participant 26*,* Manager)“And there’s a lot of really good*,* you know*,* using of social media*,* using apps*,* to reach out to children and get real time information for children of pain*,* which I think has applicability to children and families with palliative care needs. (Participant 19*,* Clinician/ Manager)*

Participants also noted how the use of technology, particularly through virtual consultations, provided better access to clinical and specialist expertise for patients.

*we are actually working on an area*,* an area of 4.5 million inhabitants and…around 200–300 kilometres altogether. So… we are sitting kind of in the middle of this area*,* and what we would like to do is to try to move patient as least as possible…if we can share an electronic data*,* electronic chart and electronic medical chart*,* I’m confident that this will be patient oriented. (Participant 5*,* Clinician/ Manager)*

#### Organisation

Building on previous themes, including the “vision” for future innovation in palliative care, participants described the organisational changes that would be required to realise these aspirations. Many described their CPC provider as a small organisation, with new models of working being required to deliver increased specialisation of roles and sites as their organisation develops and grows.

*We absolutely support the need for providers to scale. From a corporate perspective*,* joining up with other providers gets you to a whole different tier of funders*,* gives you more options to pool and specialise your staffing model. There’s no way we could have done what we have if we’d stuck to working as an individual hospice. (Participant 46*,* Clinician/ Manager)*

*We stand alone as a small organisation – we’re independent of the wider health system but that doesn’t mean we can’t find some ways to benefit from scale – whether that’s by setting up networks or collaborations or partnerships. That can be formal or informal but it can give use the scale to do much more than we can on our own (Participant 11*,* Clinician/ Manager)*

#### Culture

Participants described the need to create a culture within CPC providers that supported innovation, ensuring that novel solutions and ideas are shared and scaled appropriately. Participants outlined the key ingredients that they perceived created a culture of innovation, requiring support and resources from within the organisation.

*We need to develop a culture and institutional innovation. That is to try continuously to be better and to apply what already works*,* not just focus on the new. Data is central to this*,* but there has to be a methodology in the DNA of the organisation and of the hospice. (Participant 43*,* Manager and Innovation expert)*

*But the four major things to have change happen rapidly are…clear vision*,* dedicated change management team… specific resources for research… And then*,* fourthly*,*… you’ve got to control the communication. And you’ve got to let everybody know regularly*,* what’s happening using the data that you’ve collected*,* talking about the things that have stopped showing the progress toward the vision (Participant 42*,* Manager and Innovation expert)*

#### Place

The physical setting for the delivery of CPC was a strong theme within the data, with three prominent sub-themes. “De-medicalising the healthcare environment” was an important concept to participants, with many describing CPC as leading in this field, incorporating a strong desire to treat children and their families that feels comfortable, similar to the home environment.

*We have this saying that we want to create a home for life*,* we want to create a place that feels homely*,* where you feel secure*,* where you can actually live your life (Participant 29*,* Manager)*

This links to the second subtheme, “changing role of the physical space” – a concept which responds to the needs of users, evolving to create a physical service that best suits their needs.

*I think the key learning is that what families really need is small buildings and big community services. Because given a choice*,* they will tend to vote two thirds community care and 1/3 hospice care. Whereas in the early days of children’s hospices*,* these big buildings were built as super regional kind of structures. And of course*,* people will travel two or three counties in order to access them. But of course*,* when they do that they’re uprooted from their community (Participant 21*,* Clinician and Manager)*

The third sub-theme related to the application of “design thinking”. There was a strong sense from participants that the future of CPC must involve user-centred design, and that to truly innovate, children and their families must be included in the design process.

*We like to focus on the user experience*,* but how do you actually do it if you’re not prepared to change your methodology*,* and actually really integrating the view of patients and human beings and the families into your design process?…so really the whole process we’d like to*,* to design from the user’s perspective*,* and from the needs that they have*,* that they articulate (Participant 48*,* Clinician and Manager)*

#### Partnerships

The creation and maintenance of partnerships between CPC service providers and those around them were a clear theme within the data. Strategic partnerships with other providers were a key mechanism to leverage substantial external resources, allowing CPC’s to offset their own small scale and expand/improve the service delivered.

*you know*,* is one way that many of us have done that*,* you know*,* trying to think outside the box… one of the things was funerals*,* funerals are incredibly expensive… we went through our local funeral directors professional association meeting*,* and just*,* you know*,* introduced ourselves*,* this is what we’re doing children*,* we’re going to be dying at the house*,* we wanted to get to know the different providers in the community. So when our families asked for referrals*,* we could give them a list of*,* of funeral*,* funeral homes. Well*,* one of the funeral home directors had lost his son as a child… And from that day on*,* he provided free services for all of our children. (Participant 50*,* Clinician/ Manager)*

These partnerships were especially important within the community – relationships in which different community groups were enabled to help care for one another, ultimately improving a child/family’s quality of life.

*We have volunteers who come in and teach them directly in class*,* we have people donating books we have*,* the walls have been painted with cartoons and everything… This is just to let the children know*,* feel they’re still children are not necessarily just bed numbers or diseases (Participant 1*,* Clinician/ Manager)*

#### Leadership

The final theme within the constructed thematic framework related to the leading role CPC providers have in the future development of the sector. Three prominent subthemes emerged within this – that of advocacy, education and research.

The importance of leaders advocating for innovation and investment in CPC services was significant for participants. Championing CPC with funders and external stakeholders was described as crucial to ensure a future equitable and inclusive service.

*We need to be really aggressive in advocating for paediatric palliative care… advocacy is about knocking those doors until they open*,* as I said earlier on*,* its to define those champions. Work with them. And again*,* is to do both*,* you know*,* from the grassroot level and at the same time*,* at the top. (Participant 49*,* Clinician/ Manager)*

Participants described the need to educate a broad range of healthcare workers in CPC – given the high demand and limited supply of resources, and requirement for informed capacity building.

*we have an education programme for those people*,* which is like a responsive thing. When a patient is diagnosed*,* we can respond and provide an education package for the people who are going to work with that family (Participant 44*,* Clinician/ Manager)*

*looking into the future*,* one of the two pillars that they need to focus on the future is actually the education and training specially for healthcare professions and others to take in the lead of palliative care (Participant 4*,* Manager)*

Participants also described a lack of high-quality research within the sector (despite a vast amount of data), and the need for leadership in this area to build evidence and improve standards.

*the hospice group or community based paediatric hospice…it’s a treasure trove for learning and research that is completely untapped.………wouldn’t it be amazing if someone created infrastructure to help organise that if there was a way so that paediatric hospitals across the country were sort of similarly storing that data with sort of similar metrics (Participant 31*,* Clinician/ Manager)*

## Discussion

The thematic framework developed in this qualitative study uniquely brings together data on the most important areas for innovation within CPC service delivery, from the perspectives of a range of experts in the field. Nine key areas for innovation have been identified and brought together, which can be used by those providing CPC services as a basis from which to frame their future initiatives and service development plans. Whilst many of these areas are not themselves new, this is the first time that they have been described together. These nine themes outline key areas for innovation and improvement within CPC service delivery.

Our analysis highlights the huge potential for the development of the application of technology within CPC, to truly innovate and provide an exemplary service for children and their families worldwide. Providers can learn lessons from the use of digital technology created in response to the COVID-19 pandemic [20, 21], and actively work together to create an ongoing market for digital innovations that will enhance the service provided to children. There are enormous opportunities to leverage new technologies within the sector – currently untapped – that may enable the empowerment of children, families and staff through information, connection and flexibility [22].

Our thematic analysis demonstrates the need for CPC services to have a clear vision of what their goals and ambitions are for the future of the sector – ensuring that their services strive to deliver world class care to all. It is well established that the scale of the unmet need in CPC is vast [2] – as such, future CPC providers may wish to ensure the perspectives of all who would benefit are included in their vision.

This study highlights that CPC providers recognise the importance of their users’ needs and opinions when developing and innovating in this sector. This compliments existing research demonstrating that children are able to engage in the design of patient-centred outcome measures [30], and that this work must be holistic – encompassing spiritual and existential concerns as well as clinical needs [31]. User-centred design must be adopted as the participatory methodology at the heart of all future planning and development. To achieve this in a meaningful way, CPC providers must foster and cultivate a culture where innovation is encouraged, expected and shared. This will facilitate the global scale of successful innovation and minimise the siloed working that can occur within the sector at present.

Building upon a culture of innovation, insights from our thematic analysis re-emphasise the importance of integration across the sector. This was described by participants, and corresponds with the WHO’s conceptual model for the development of CPC [32]. This may take the form of integrated care – with the seamless delivery of a diverse and holistic range of services that meet the full complement of users’ needs. The need for integration is also reflected in the way that multiple healthcare services, professionals and community initiatives must operate together to create an innovative and ever-evolving provision of CPC in a range of environments. CPC is a sector that remains nascent – as such leaders must champion and integrate external facing activities such as education and research in order to grow in size, stature and sustainability. Whilst this research field is growing [32], evidence remains limited and work should continue to take place to further develop this. This need for ongoing research currently limits the ability of CPC to be truly innovative – to be successful innovation must be built upon a known evidence base for the effectiveness of the current service and iterated to reflect the impact of adjustments and improvements.

### Integration with previous work

The findings of this study are supported by existing literature. Our participants’ perspectives corroborate previous evaluations of the potential benefit of digital health interventions [33], taking this one step further by presenting this field as the key area for future innovation in the sector. Our thematic framework showcases the need for integrated, seamless and holistic care – something acknowledged in the literature as being essential for CPC [34, 35], and demonstrates where innovation would be most useful to further the benefits of this. Participants in our study strongly advocated for the application of user-centred design when innovating in the sector. This is supported by the successful application of user-centred design and person-centred communication in both palliative care and the paediatric population [36, 37]. Whilst this methodology is gaining traction, there is still much work to do to ensure all future innovation takes place by keeping patients at the centre of the process.

### Transferability

Given the global nature of this study and representation of CPC professionals and experts within the participant sample, we believe the findings of our qualitative analysis will be generalisable to all those working in CPC worldwide, especially those looking to develop and innovate their provision. We acknowledge that the need for CPC may have individual nuances and therefore may vary across different countries. The thematic framework created in our analysis houses nine themes that can be considered when looking to understand what innovation means in the context of CPC and where opportunities for future innovation lie. It provides another lens for viewing innovation in CPC through, which complements existing tools such as the WHO conceptual model [32] and other global initiatives such as the World Health Assembly resolution on palliative care [38]. In addition, we encourage the nine themes evident in our data to be explored in the context of a local review of the needs and challenges within each CPC service, as this may highlight how different aspects of these data may be prioritised according to context.

### Limitations

There are several limitations within this study. Whilst we strived to include a breadth of participant experience through purposive sampling, the study is at risk of participation bias – those with an interest in innovation in CPC (or a perception that they were innovative in their own practice) may have been more likely to take part in the study, as may those who felt frustrated at a lack of resources or ability to innovate within their service. There may also have been a risk of social desirability bias – with participants keen to discuss their service in a positive light. We did not conduct an analysis according to experience type (e.g. clinical vs. managerial), and as such some nuances in the participant’s perspectives regarding the need and possible mechanisms of future innovation may have been missed.

Not all the participants in this study had extensive experience in the field of CPC – this could be viewed as a limitation as they may not have been aware of existing work in the field and what is currently innovative. However, in the context of innovation this might also be viewed as a strength – with these participants not being limited by their previous experience or knowledge of resource constraints.

All interviews were conducted in English – this may have excluded perspectives and participants who did not meet this inclusion criteria.

Finally, our participant sample was limited predominantly to those with direct experience of working in or advocating for CPC, which may have limited the predictions and aspirations for future innovations within the sector identified in this study. It may be that those outside the sector “looking in”, may have more clearly identified opportunities for innovation. We also acknowledge that patient and family perspectives are crucial – whilst these voices were represented within our participant sample to a degree in the form of advocates, further work can be done to incorporate these voices.

### Opportunities for future work

It would be interesting to build upon the results of this analysis – recommendations for future research include conducting a quantitative evaluation of the presence of innovation in the sector and its impact. It would be of benefit to identify the key areas within the framework that are important to children and their families, through a review of the framework and subsequent amendments as appropriate.

Future work could also investigate current areas of technology and digital innovation being used within CPC, evaluating how these could be scaled and developed, working to fund targeted innovations (using user-centred design), with a robust evaluation and modelling of their potential impact.

We present a general framework for innovation in this paper, future work could be more targeted to evaluate which specific interventions might be valuable when trying to solve core issues within the sector – such as a lack of funding and limited capacity to meet the needs of the population. This evaluation could incorporate a mix of proven intervention and potential interventions that may hold promise – such as the use of artificial intelligence.

Finally, it is important to look at how our framework fits in with the existing WHO conceptual model [32], and how it can be used to help overcome the barriers and challenges in CPC development.

## Conclusions

Innovation is vital in improving access to care, driving ongoing excellence, and ensuring the quality of care offered is of a high standard. To address the ongoing unmet need in the provision for high quality CPC worldwide, the sector needs innovations that can lead to a tangible step change to the provision of care. This must incorporate both an improvement in the care that is delivered and ensuring that access to this care is provided to all who need it. This study provides a basis for the direction and needs of innovation that leaders and policy makers can look to when determining how to improve the current service, engage and empower those who use it and contribute to achieving high quality CPC for all children and their families who need it.

## Electronic supplementary material

Below is the link to the electronic supplementary material.

Supplementary Material 1

Supplementary Material 2

## Data Availability

Data is provided within the manuscript. To protect participants privacy recordings of interviews cannot be shared publicly. Qualitative data in the form of interview transcripts are available from the corresponding author upon reasonable request.
