# Supplementary material for: What are the greatest opportunities for innovation to improve access to and quality of palliative care services to children? A qualitative interview study
Source: BMC Palliat Care. 2025 Jul 2;24:184. doi: 10.1186/s12904-025-01837-9 (PMC12220075; doi:10.1186/s12904-025-01837-9)
Supplement: Supplementary file 2 — Supplementary Material 2 [file 12904_2025_1837_MOESM2_ESM.pdf]

<u>Interview template for GTF Hospice of the Future Project</u>

User Note: The following is a guide to inform discussion with identified experts in paediatric palliative care and/or health innovation. It is not intended to be a script or prescriptive set of questions, but rather a set of prompts to aid the discussion and ensure key points are not missed off. Questions in bold are the priority domains, those beneath in bullet points are prompts – questions to ask to probe further into the interviewee's answers if what they have said so far doesn't cover what we're asking or they need additional description.

Context to share with interviewees at start of the interview

The framing of the interview is important to avoid only discussing the current state of paediatric palliative care; rather, we want to emphasize our interest in the future

- This is a study being produced by the Imperial College of London's Institute for Global Health Innovation, as a general global good. We also plan to publish an academic journal article emerging specifically from the interview findings.
- The official name of the study is the "Children's Hospice of the Future," but really it's about a positive vision of paediatric palliative care more generally, and particularly the most promising avenues for future innovation in the sector.
- The overarching goal of the study is to provide a detailed blueprint for a world-class children's hospice/palliative care center
    - Includes considerations for high-, middle- and low-income countries and adapting such a blueprint to low-resource settings
    - Includes best practice benchmarks and case studies from around the world, as well as aspirational goals for children's hospice
    - We are using a wide lens as to the potential domains of innovation – not just service delivery but organizational and funding models, technology, governance and the wider factors that would support an ongoing culture of innovation within children's palliative care providers more generally.

Explain that we will be taking notes and some quotes from what they say, obtain permission to record the interview, and give the interviewee a chance to ask any questions and clarify the scope of the interview.

**1) What have been the biggest areas of improvement and innovation in paediatric palliative care, operationally and organizationally, over the course of your work in the sector?**
• How might a child or family's experience be different now from 20-30 years ago?<br>•

**2) What do you see as the most promising areas of future improvement and innovation in the field?**
• Are there innovations you've seen in other areas of healthcare e.g. children's hospitals or adult hospices that you think would have particular potential if transferred and adapted across to PPC?
**3) How has the Covid-19 pandemic affected your work? How have you shifted your ways of working throughout this "new normal," and what do you expect to have a lasting impact on the field?**
**4) To what extent have palliative care programs shifted care to the home, and how much further does home care have to go if looking at the future of world class PPC?**
• What technologies might further spur this shift?<br>• What opportunities do you see for care to be provided at a community level?<br>• In contexts where there is demand from families for respite care, how can future PPC programs best meet this need? What proportion of these services can be provided in the home?

**5) Where do you see technology having the largest impact on the future of PPC?**
• What do you think about opportunities for technology to support parents caring for their children? (Eg: AI-powered platforms such as chatbots that serve as intelligent supportive tools for parents to turn to with commonly asked questions)<br>• What about technologies to support care for paediatric patients with limited motor skills and/or verbal communication abilities? (Eg: virtual games for children with limited motor skills to interact with, or platforms for children with limited verbal communication abilities to communicate preferences or choices)
**6) How have mental health support structures evolved within PPC, and have you seen any examples of excellence or ideas that have potential for scale-up?**
• How can PPC teams best support families post bereavement?<br>• How can PPC teams best support their own staff?<br>• What non-traditional staff might be included in a future interdisciplinary PPC team?
**7) How can future PPC providers best coordinate care for complex cases?**
**8) What do you think the future of staffing child palliative care programs, in terms of skill mix and training?**
• Would task-shifting from specialist doctors to GPs/NPs or less specialized providers be useful for future PPC programs?<br>• Where might volunteers and CHWs be of most use?

• What non-medical, non-traditional staff (such as social workers, therapists, lawyers, etc.) will be important include in future PPC teams?<br>• What new cadres of workers might emerge in the future of PPC programs?
**9) What do you think about the future of the physical building of the hospice? Will future hospice buildings look any different than the traditional model?**
**10) What additional types of support services have you seen being offered at children's hospices that are outside the scope of traditional PPC services that you think are most useful?**
• What new services do you think should be included within the scope of future PPC services?
**11) What, if any, emerging trends do you see in the funding models for PPC? Have there been successful pushes to integrate PPC into public health funding in your experience?**

**12) How would you structure a children's hospice at a governance and managerial level that wanted to promote innovation and continuous improvement in the way that services are delivered?**
**13) Of the innovations we've discussed today, have you seen any patterns or potential platforms through which scale up has/could happen?**
**14) Standing back from everything we've spoken about, are there any other key issues, points or examples we've not mentioned that you think are important?**

**If we’re looking for further interviews, who would you recommend?**
**Any other comments?**
